# Vegetation Cover Dynamics and Resilience to Climatic and Hydrological Disturbances in Seasonal Floodplain: The Effects of Hydrological Connectivity

**DOI:** 10.3389/fpls.2017.02196

**Published:** 2017-12-22

**Authors:** Linlu Shi, Yuyu Wang, Yifei Jia, Cai Lu, Guangchun Lei, Li Wen

**Affiliations:** ^1^School of Nature Conservation, Beijing Forestry University, Beijing, China; ^2^Water, Wetlands and Coastal Science Branch, NSW Office of Environment and Heritage, Sydney, NSW, Australia

**Keywords:** wet meadow, ecological resilience and resistance, hydrological connectivity, EVI, Three Gorges Dam, Poyang Lake

## Abstract

Floodplain wetlands are valuable ecosystems for maintaining biodiversity, but are vulnerable to hydrological modification and climatic extremes. The floodplain wetlands in the middle Yangtze region are biodiversity hotspots, particularly important for wintering migratory waterbirds. In recent years, extremely low winter water level events frequently occurred in the middle Yangtze River. The hydrological droughts greatly impacted the development and distribution of the wet meadows, one of the most important ecological components in the floodplains, which is vital for the survival of many migratory waterbirds wintering in the Yangtze region. To effectively manage the wet meadows, it is critical to pinpoint the drivers for their deterioration. In this study, we assessed the effects of hydrological connectivity on the ecological stability of wet meadow in Poyang Lake for the period of 2000 to 2016. We used the time series of MODIS EVI (Enhanced Vegetation Index) as a proxy for productivity to infer the ecological stability of wet meadows in terms of resistance and resilience. Our results showed that (1) the wet meadows developed in freely connected lakes had significantly higher resilience; (2) wet meadows colonizing controlled lakes had higher resistance to water level anomalies; (3) there was no difference in the resistance to rainfall anomaly between the two types of lakes; (4) the wet meadow in freely connected lakes might approach a tipping point and a regime shift might be imminent. Our findings suggest that adaptive management at regional- (i.e., operation of Three Gorges Dam) and site-scale (e.g., regulating sand mining) are needed to safeguard the long-term ecological stability of the system, which in term has strong implications for local, regional and global biodiversity conservation.

## Introduction

Riparian floodplains are areas adjacent to rivers that are periodically flooded (Junk, 1989). They are hydrologically important (e.g., flood mitigation), environmentally sensitive (e.g., regulating climate), and ecologically productive areas that perform many natural functions and services (Costanza et al., 1997; Dudgeon et al., 2006; Acreman and Ferguson, 2010). The high productivity is particularly important to sustain regional and global biodiversity (Shiel et al., 1998; Selwood et al., 2017). Despite their high productivity and vital ecological functions and services, floodplains are among the most endangered landform types worldwide (Millennium Ecosystem Assessment, 2005; Nilsson et al., 2005); and hydrological alternation is one of the most excessive anthropogenic pressures threating the ecological integrity of floodplains (Graf, 2006; Arias et al., 2014; Cochrane et al., 2014; Zarfl et al., 2015).

Ecosystem function and service are closely linked with ecological stability, which can be characterized as the persistence near or close to an equilibrium state (i.e., resilience, Holling, 1973) or the tendency of returning to the reference state after a temporary disturbance (i.e., resistance, Gunderson, 2000). Resistance describes the ability of the ecosystem to absorb a disturbance and maintain its pre-disturbance state (Ruijven and Berendse, 2010; Vogel et al., 2012). Similarly, resilience is defined as the speed of recovery to the equilibrium state after a disturbance event (engineering resilience) or the amount of disturbance that is needed for a system to switch equilibrium state (ecological resilience) (Holling, 1996). Long-term measurements of ecological state are useful to calculate resistance and resilience (De Keersmaecker et al., 2015; Wen and Saintilan, 2015; Ivits et al., 2016; Verbesselt et al., 2016), and changes in the two metrics provide an indication/early warning of imminent irreversible changes in equilibrium states (De Keersmaecker et al., 2016).

In the recent decade, ecological resilience/resistance has gained tremendous stock in natural resource management policy (Benson and Garmestani, 2011). Many studies in terrestrial and aquatic ecosystems has shown that abrupt ecological changes often occur in response to natural and anthropogenic perturbances (Folke et al., 2004). As such, an understanding of the underlying processes is desirable for successful management (Seastedt et al., 2008). Vegetation communities in floodplains provide a unique natural laboratory to explore concepts of resistance and resilience in relation to climatic and hydrological perturbations (Batt et al., 2013). In floodplains, recruitment opportunities, regeneration, growth rate, interspecies competition and survival are all associated with flooding regime, including the timing, extent, duration and depth of flooding (Bornette and Puijalon, 2011). River flow regime therefore interact with floodplain morphology to create a spatial matrix of niches with distinct hydrological conditions, which subsequently support an array of vegetation communities (e.g., submerged, emerged, woody, and forest). Differences in species adaptive strategies, thresholds of survival, and competitive advantage create patterns in vegetation community distribution along flooding gradients broadly repeated across floodplains and catchments within climatic zones (Junk, 1989; Horner et al., 2012). This association between vegetation and flooding history has raised concerns about the effects of water resource development, river regulation, and climate change on the integrity of floodplain vegetation (Bino et al., 2015).

The middle Yangtze River system is rich in floodplains, among which Dongting Lake and Poyang Lake are the two most predominant ecosystems with free flow connections with Yangtze River (Sun et al., 2012; Lai et al., 2014). Like other large river systems such as the Mississippi River floodplain (Phelps et al., 2015), Pantanal floodplain (Fantin-Cruz et al., 2016), and Amazon River floodplain (Arnesen et al., 2013), the Yangtze floodplains have experienced significant changes due to hydrologic modifications for navigation, flood control, hydro-power, and other localized regulation structure (Yang et al., 2011). Three Gorges Dam (TGD), the world's largest dam, which started to impound water in 2003, and fully operational since 2006 (www.3g.gov.cn), arguably has the largest impacts on flow and sediment regimes (Yuan et al., 2015). Since the operation of the TGD, many researches have showed that the inundation regimes and the distribution patterns of wetland habitats in floodplains changed dramatically (e.g., Du et al., 2011; Xie et al., 2015; Jing et al., 2017), which in turn has detrimental impacts on its ecological function as wildlife habitats such as fish (Turvey et al., 2010) and migratory birds (Wang et al., 2013; Jia et al., 2016). Despite the continuous debate on causality, these hydrological and ecological changes were linked to the TGD (Mei et al., 2015, 2016; Liu et al., 2016) and the extensive sand mining (Ye et al., 2014). However, there is a knowledge gap on the ecological stability of the vegetation communities in floodplains of the middle Yangtze region. Considering the area is the only wintering ground for a number of endangered species as Siberian Crane (*Leucogeranus leucogeranus*) and the oriental white stork (*Ciconia boyciana*) (Wang et al., 2013), ecological stability is vital for their survival. Critical questions urgently need to be answered for biodiversity conservation in the middle Yangtze region. For example, is the observed expansion of grasslands (Mei et al., 2015; Jing et al., 2017) reversible? is it a gradual environmental change or the system is moving toward a new regime when a critical threshold is exceeded (Dakos et al., 2015)? What's the recovery potential of the system? In this study, we adopted a quantitively analytic framework to tackle these issues in terms of ecological resilience and resistance.

This study focused on wet meadows in Poyang Lake, a large seasonal floodplain in humid subtropical climatic zone. The wet meadows are largely monospecies dominated by *Carex* spp. (Guan et al., 2014; Xie et al., 2015), which is the main foraging ground of many migratory waterfowls during low water (Guan et al., 2016), and fish spawning and feeding site when water level is high (Xie et al., 2015). Within the Lake, there are three major types of sub-lake in terms of hydrological regulation (Xia et al., 2016): freely connected lakes with no manmade hydrological structure; partially regulated ones with low level bank enforcements, and are normally equipped with sluices to keep water high in winter; and isolated one with high artificial levee banks, they are totally separated from the rest of the lake with very limited water exchange. Generally, wet meadow is absent from isolated lakes, therefore, they are not included in this study. The regulation gradients enable us to compare the effects of hydrological regulation on the ecological stability of wet meadows, which could provide practical guidelines for wetland conservation in Poyang, such as building hydraulic structure on the outlet channel to modify the dynamic links between Yangtze River and Poyang Lake (Zhang et al., 2014).

We developed autoregressive modeling (AR) to explore the stability of wet meadows using long-term (2000–2016) deseasonalized and detrended time series of vegetation dynamics anomalies (Verbesselt et al., 2016). We also investigated the effects of hydrological and climatic anomalies on the temporal dynamics of wet meadow by including them in the AR models as exogenous variables. The ecological state variable is represented by biomass productivity, which is in turn approximated using the enhanced vegetation index (EVI) (Huete et al., 2002). The aim of study was first to compare the ecological stability of wet meadows in connected and controlled lakes. Secondly, we evaluated the relative importance of hydrological and climatic variables on the stability of the wet meadow.

## Methods

### Study site

**Figure 1:**
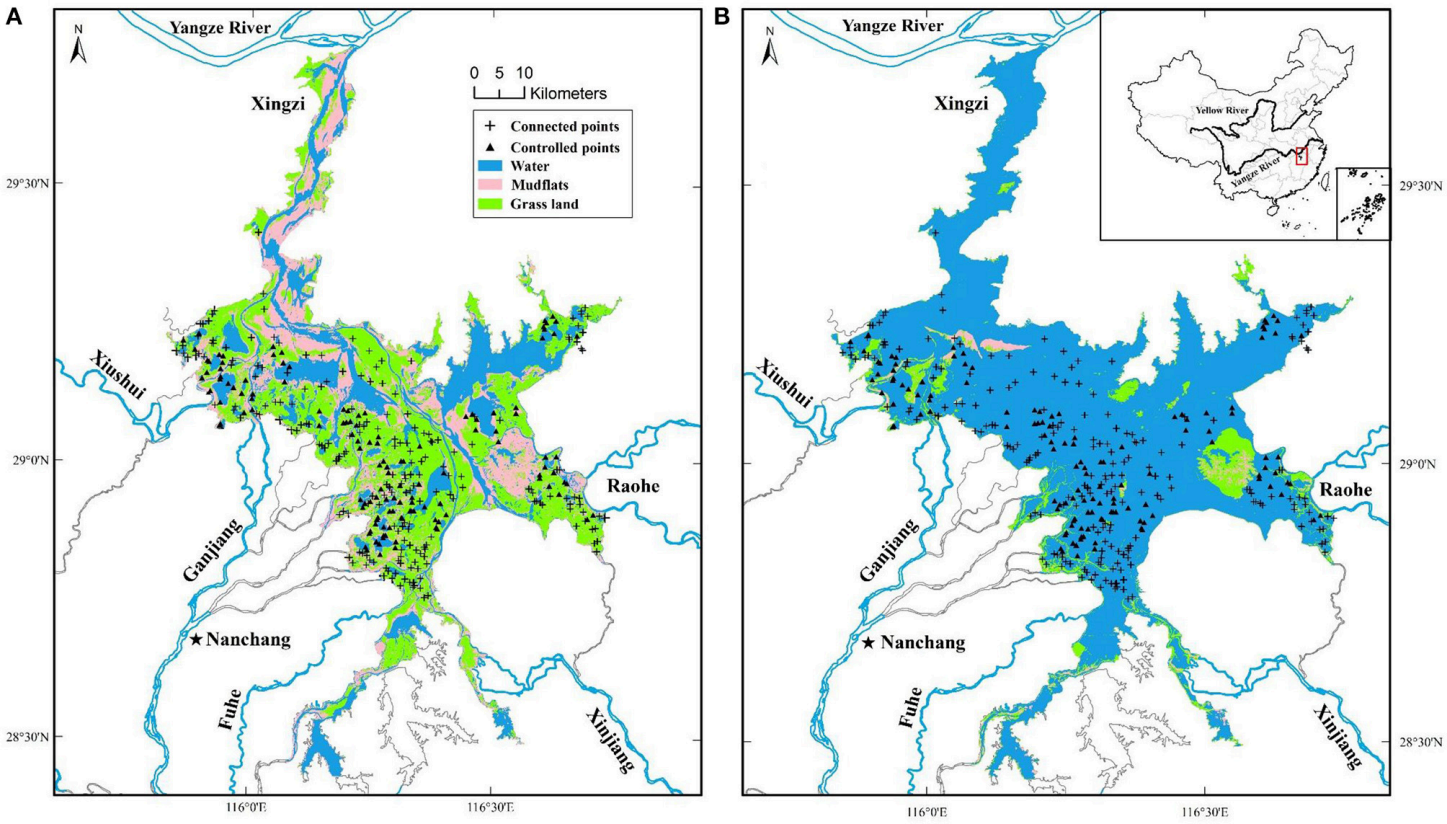
Location of Poyang Lake at dry **(A)** and wet **(B)** seasons. Sampling points were randomly selected with the restraint of 300 m apart.

Poyang Lake (28°22′–29°45′N, 115°47′–116°45′E, Figure 1) is located at the southern bank of the Yangtze River. It is naturally connected with the Yangtze via a wide channel (average about 4,500 m, and over 1,000 m at the confluence). With an area of nearly 4,000 km^2^ during summer high water period (Shankman et al., 2010), it is among the largest freshwater wetland complexes in Asia. The prevailing climate in Poyang region is humid subtropical, characterized by hot and humid summers with frequent storms, and frequently dry winters.

The inundation regimes and water level fluctuations have clear seasonal patterns, and are largely controlled by the water balance of the five major tributaries (Figure 1) and the Yangtze River. During high water seasons, the system is whole water body (Figure 1B). In the low water level winters, the surface water area normally shrinks to <1,000 km^2^ (Dronova et al., 2011; Feng et al., 2012), and the Lake system becomes a complex ensemble of hydrologically distinct streams and sub-lakes interspersed with mudflats and rapidly colonized wet meadows (main species are *Carex* spp.) (Figure 1A), providing vital wintering habitats for a range of migratory waterbirds.

Many of the sub-lakes are managed for fishery (Zhang and Li, 2007). Based on the levels of hydrological control, the sub-lakes can be grouped into three classes, namely, isolated, partially regulated, and freely connected (Xia et al., 2016). The isolated lakes have high manmade banks and are totally separated from the main lake with limited surface water exchange. The partially regulate lakes have limited vertical bank enhancement but the gaps in natural banks were filled and equipped with sluices, which are functional only in dry seasons when the water level is low. The freely connected lakes have no regulation and represent the natural hydrological regime. In this study, as wet meadows are rare in isolated lakes, we focused on the partially controlled (referred as controlled) and freely connected (referred as connected) sub-lakes.

Within each of the sub-lakes, we created 10–20 random points depending on the size of the sub-lake. The shortest distance between any two randomly placed points was restricted to be greater than 300 m to ensure a unique time series. In addition, there are large number of patches of wet meadow in flat areas which have no obvious bank (natural or artificial). Random points placed in these flats were classified as connected. We then used the datasets of spatial point to extract the 16-day vegetation index (see below).

### Data sources and data preparation

#### EVI time series

The normalized difference of vegetation index (NDVI, Kriegler et al., 1969) and EVI (Huete et al., 2002) are commonly used as a proxy of plant photosynthetic activity, biomass and productivity to assess vegetation dynamics from space (Garroutte et al., 2016). In the subtropical Poyang region, wet meadow can rapidly colonize newly exposed lake bed and form dense monospecific patches (e.g., *Carex* sp., Jing et al., 2017), and the NDVI values can be saturated (Huete et al., 2002), therefore, we used the EVI to investigate the vegetation response in this study. While enhancing sensitivity to vegetation density, the EVI also reduces the variation in canopy background signal as well as the atmosphere influences (Huete et al., 2006). With the spatial sampling points, we extracted 400 time series of the 16-day EVI time series (2000–2016) from the U.S. Geological Survey (USGS) Earth Resources Observation and Science Center (EROS, http://LPDAAC.usgs.gov). As the EVI was used as a surrogate for primary productivity and biomass (Sims et al., 2006; Glenn et al., 2008), we excluded time series with maximum value < 0.1 (very low EVI value might corresponding the areas of water and/or bare ground of mudflats with no wet meadow developed), and resulting in a total of 294 time series (180 in connected lakes and 114 in controlled ones).

#### Trends, seasonality and anomaly of EVI

To obtain an accurate and un-biased estimate of ecological resilience and resistance, time series need to be stationary without trends and seasonal patterns (Santer et al., 2000; Dakos et al., 2009). A primarily screening indicated that the raw time series of EVI displayed clear seasonality and trend, and there is evidence that the seasonality (i.e., intra-annual variation) increased as well (an example was presented in Figure S1). To account the trends and seasonality, especially the variation in seasonality, we adopted the loess (Local Polynomial Regression) smoothing method by Cleveland et al. (1990) to decompose the raw time series into three different time series:

(1) $$\begin{array}{r} Y_{t}=S_{t}+T_{t}+R_{t}\text{ for }t=1,2...,n \end{array}$$

where *Y*_*t*_ is the raw observation data at time *t*, and *T*_*t*_, *S*_*t*_ and *R*_*t*_ are the trend, seasonal, and remainder variation components of the data. The filtered time series *R*_*t*_ (i.e., de-trended and deseasonalized EVI) were then used for further analysis (Verbesselt et al., 2016).

We tested and validated the stationarity of the filtered EVI time series using the Kwiatkowski-Phillips-Schmidt-Shin (KPSS) test (Kwiatkowski et al., 1992), and all the resultant time series passed the KPSS test at 0.05 significant level.

#### Environmental variables

Daily water level at Xingzi and climatic (rainfall and temperature) at Nachang for the period of 1999 to 2016 were obtained from the Changjiang Water Resources Commission (http://www.cjh.com.cn) and China Meteorological Data Service Center (http://data.cma.cn/en), respectively. To match the frequency of EVI, we aggregated the daily time series to the same frequency of EVI (i.e., 23 samples/year). As with EVI time series, the environmental variables displayed clear seasonality, and to a less degree, trends as well (Figures S1–S3). Therefore, the water level and climatic variables were decomposed used the same approach described above. We checked the correlation between the filtered environment variables using Pearson's coefficient, and the linearity among them is low (Pearson's *r* were −0.22, −0.12, and 0.17 for water level—temperature, rainfall—temperature, and water level—rainfall, respectively). In addition, we found that there was a lagged response of vegetation to environmental perturbations, and used the one-step ahead time series of hydrological and climatic time series in the modeling.

### Ecological stability: resistance and resilience

The vegetation cover dynamics and resilience to hydrological and climatic disturbance was explored using dynamic linear regression models with the filtered water level, rainfall and temperature as exogenous factors. Regression models in which a time-lagged dependent variable is used as an additional predictor variable are often referred to as dynamic linear regression models—also known as autoregressive (AR) models. AR models are commonly used for the analysis and forecasting of time series data in economics (McLeod et al., 2012) and hydrology and climate (Anderson, 2011). The lagged dependent variable introduces a temporal component into the model, so that the EVI at a given time step is also a function of the EVI of the previous time step in the time series as:

(2) $$\begin{array}{r} y_{t}=\varphi y_{\left(t-1\right)}+\beta X_{t}+\varepsilon_{t\;},\;t=1,\;2,\;3,\;\ldots ,\;n \end{array}$$

where at time *t*, *y*_*t*_ is the filtered EVI; *y*_(*t*−1)_ is the filtered EVI value at the previous time step, *X*_*t*_ is a vector of the regressors including the intercept, the filtered water level, rainfall and temperature at time step *t*−1; φ is the autocorrelation coefficient, β is the coefficients vector; and ε_*t*_ is the white noise i.i.d (0, σ2).

To avoid spatial correlation, we fitted AR model for individual EVI time series (i.e., a total of 294 models were developed). In addition, we standardized all the anomaly time series prior to model fitting (Verbesselt et al., 2016) to allow the direct comparison of impacts of rainfall and water level (Wen et al., 2011). We extracted the model coefficients and used them to infer the ecological resilience and resistance of floodplain wet meadows to hydrological and climatic perturbations as proposed by De Keersmaecker et al. (2015). By definition, the temporal autocorrelation (TAC) coefficient φ, which describes how the current EVI is influenced by its previous values (i.e., time series memory), provides an indicator of ecological resilience (Ives, 1995; Dakos et al., 2012). TAC is in the range of −1 to 1, and large absolute values indicate a slow return to equilibrium or low resilience (Wen and Saintilan, 2015; De Keersmaecker et al., 2016; Verbesselt et al., 2016). The regression coefficients for water level, rainfall and temperature in the β vector, are related to the resistance to disturbances. If the coefficients are significant (i.e., *p* < 0.05) and large in absolute terms, the resistance is consequently low (De Keersmaecker et al., 2016).

### Effect of landscape position on the stability of vegetation community

We compared ecological stability of wet meadows in terms of resilience and resistance between connected and controlled lakes using permutation *t*-test, and reported the exact *p*-values estimated by 10,000 Monte Carlo resampling (Fay and Shaw, 2010). We chose to use the robust permutation *t*-test as the data were not balanced, and more importantly, violated the assumption of normality distribution.

All modeling and statistical analysis were done in R (version 3.1.1; R Development Core Team, 2014).

## Results

### Long-term trends and seasonality in EVI

**Figure 2:**
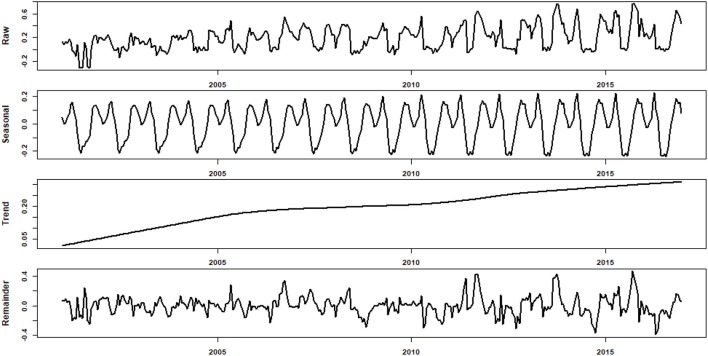
The raw, decomposed seasonal, trend, and remainder of a typical EVI time series at a typical connected site. The EVI continuously increased for the entire study period, and the seasonality also showed an increasing trend.

**Figure 3:**
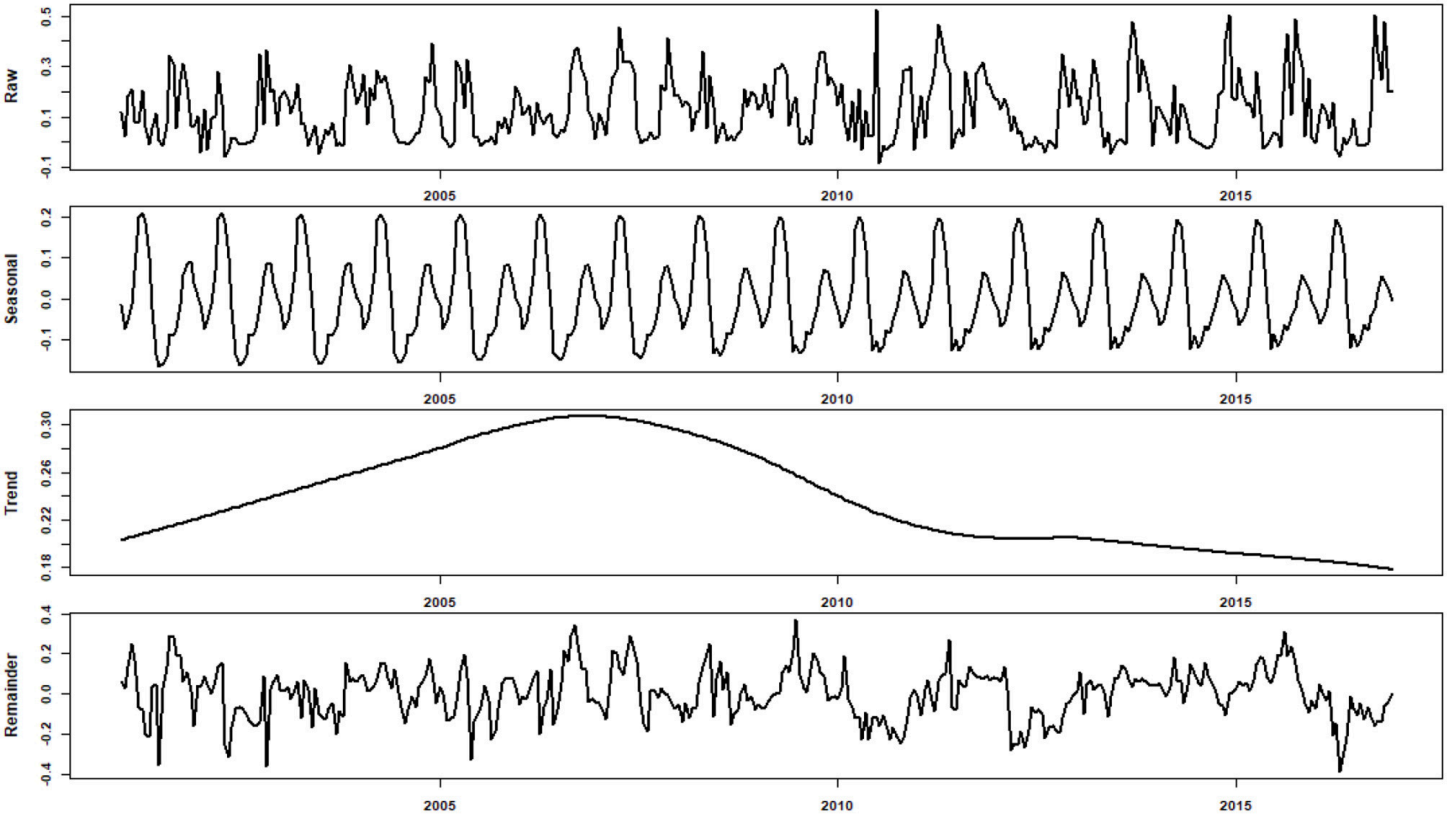
An example of the raw, decomposed seasonal, trend, and remainder of EVI time series at controlled site. The seasonality was stable for the study period. The trend showed a distinct pattern comparing to connected sites: the EVI increased continuously till 2006, since which a decreasing trend was evident although the decreasing rate was much smaller since 2003.

**Figure 4:**
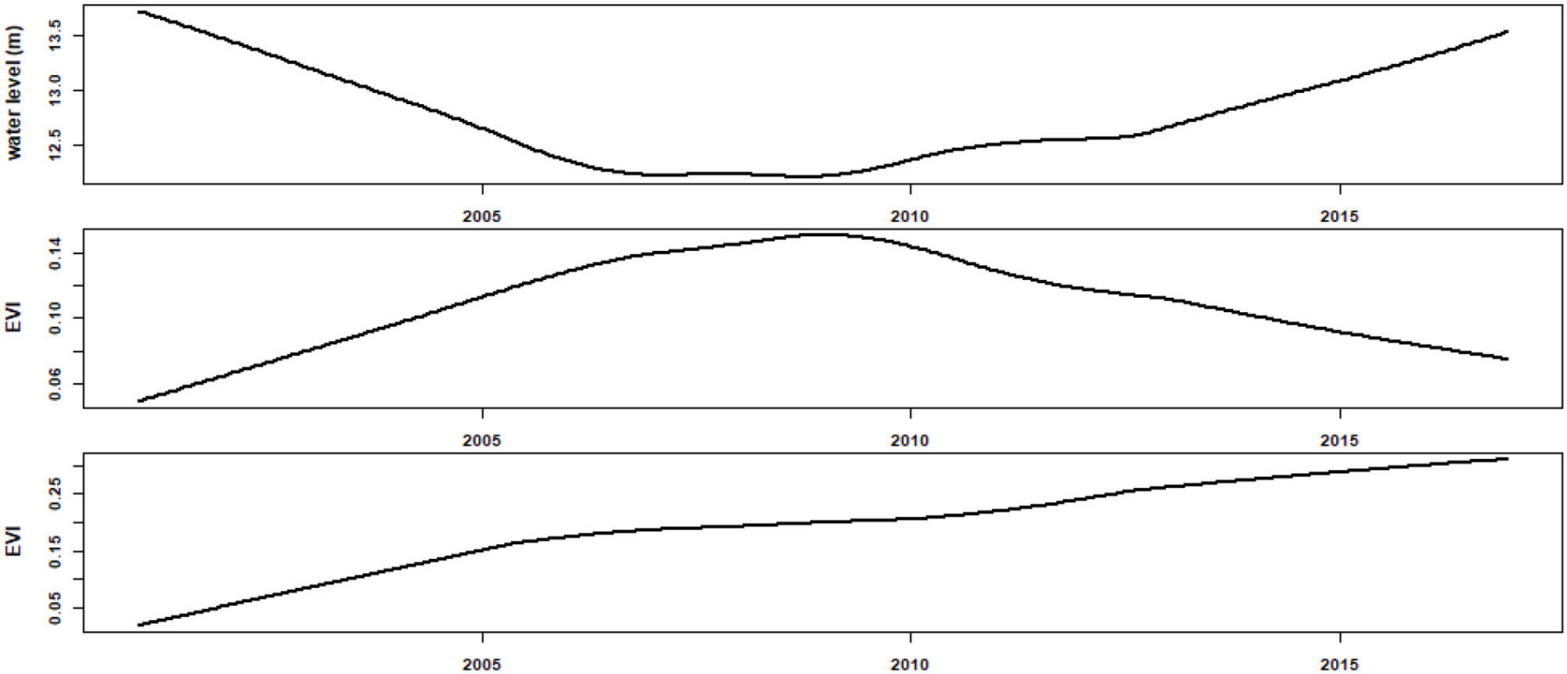
The trend of water level at Xingzi **(upper)**, examples of EVI trend of the wet meadows in controlled **(middle)**, and connected sub-lakes **(lower)**, showing the opposite but synchronous trends between water level and EVI for controlled sub-lakes.

Most of the EVI time series at connected sites showed clear intra-annual variation (i.e., seasonality), and there were evidences indicating that the seasonality increased over the study period (Figure 2). Furthermore, the EVI increased continuously for the study period. Most of the EVI time series at controlled sites, however, showed distinct dynamics compared with those at connected sites (Figure 3). In general, the wet meadows in the two classes of sub-lake had distinct trends during the study periods. While the EVI values of wet meadow in the free-connected lakes increased monotonically, those in the controlled lakes had a unimodal (“hump-back”) shape (Figure 4). The productivity of wet meadows within partially regulated lakes showed an increasing trend to around Nov 2006, but the elevated EVI values decreased gradually to the end of the study period. The opposite but synchronous trends between water level at Xingzi and the EVI in controlled sub-lakes indicated the negative impacts of water level on the development of wet meadows. However, this relationship was absent for freely-connected sub-lakes (Figure 4).

An increase in seasonality was found in many time series (>65%) of EVI from the connected lakes while the majority from the controlled lakes were relatively stable (an example was presented in Figure S4).

### Ecological resistance/resilience to environmental variations

**Figure 5:**
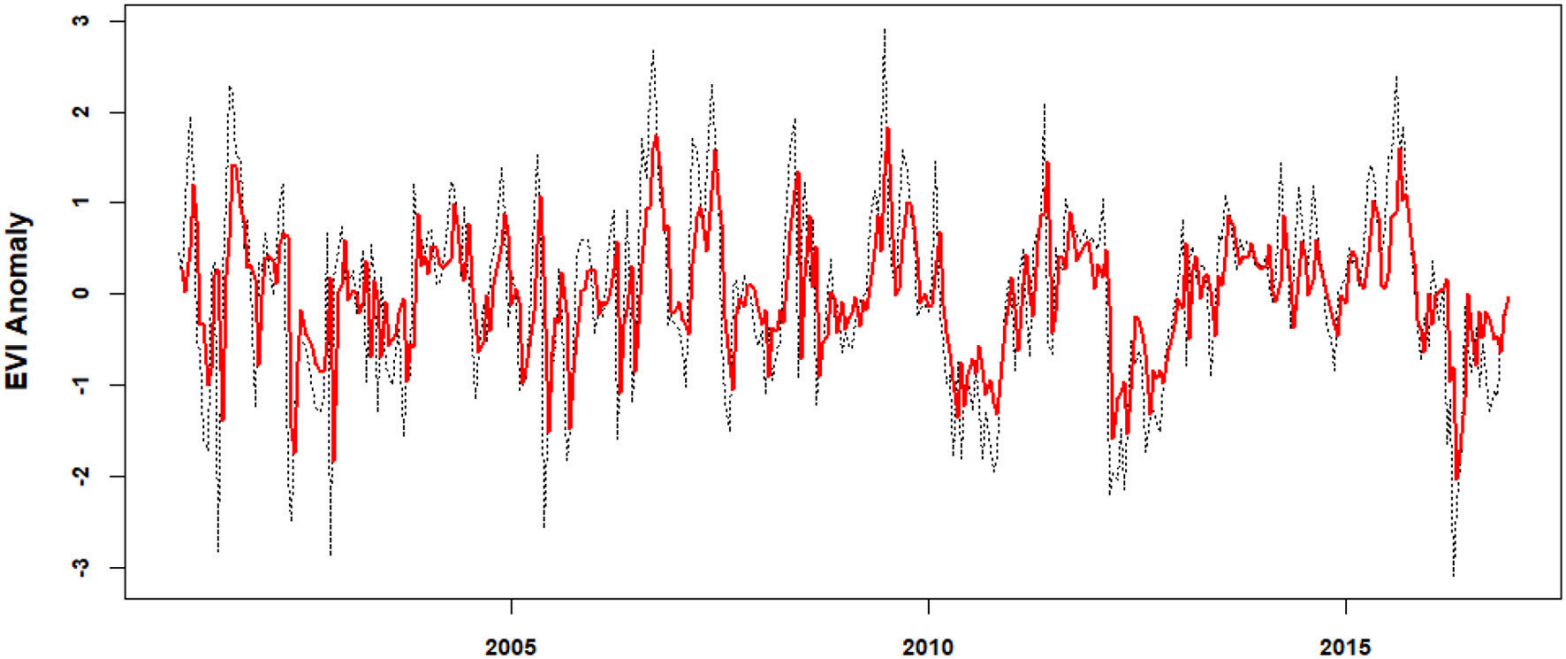
An example of randomly selected autoregressive model (i.e., no 295, red line) for the filtered EVI time series. Dotted line is the filtered EVI anomaly.

The AR models preformed relatively well for the majority of filtered EVI time series (the adjusted *R*^2^ ranges from 0.224 to 0.573), and the fitted models generally traced the dynamics of time series well although model errors for extreme values were large (Figure 5).

Temperature was significant (i.e., *p* < 0.05) in only four of the 294 AR models. We found these four points were located at the lake edge adjacent to cropland. The EVI values might also include signal from crops. Therefore, we didn't include the four time series in further analysis, and temperature was omitted from the AR models.

**Figure 6:**
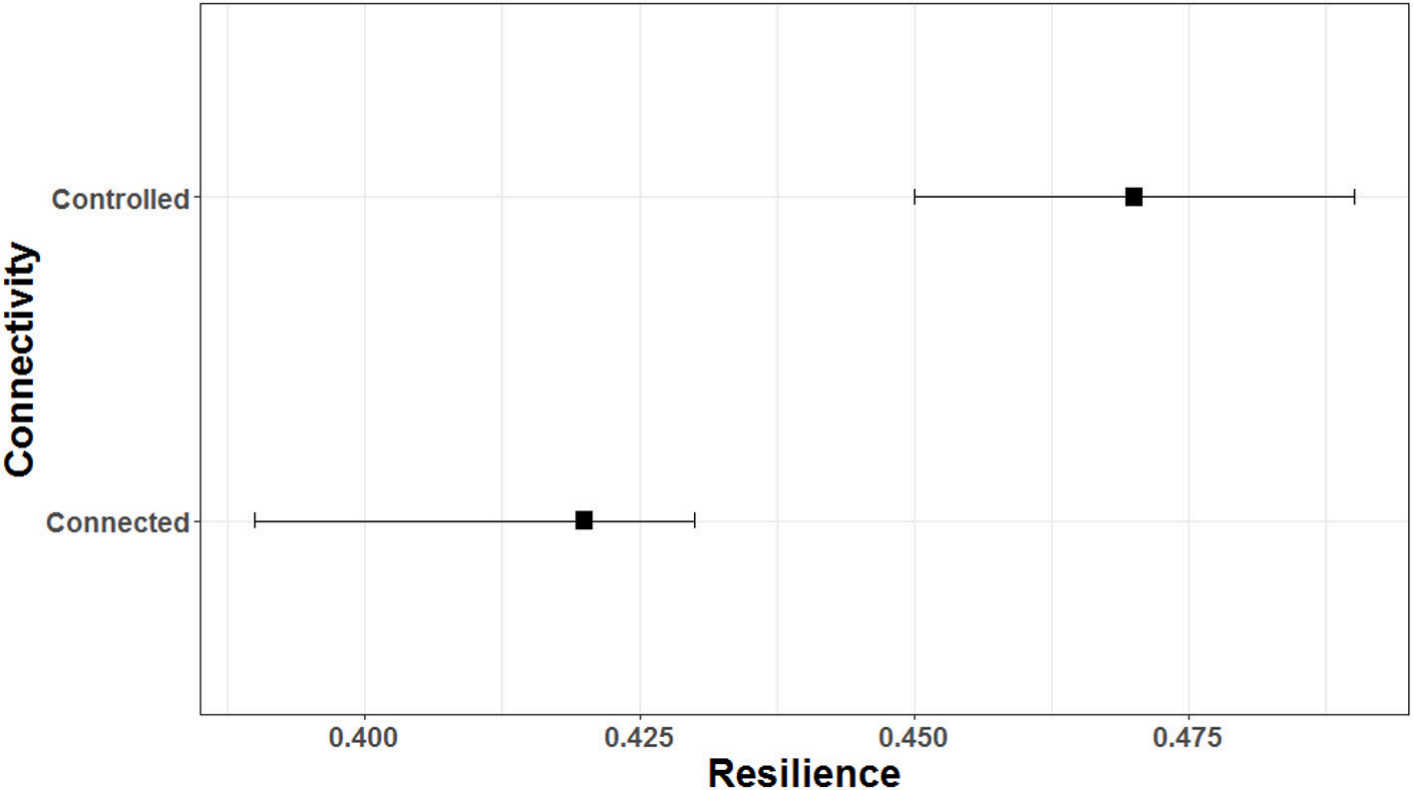
Temporal autocorrelation coefficients of the filtered EVI time series (2000–2016). Squares are mean and bars are 95% confident intervals based on 10,000 bootstrap resampling. There is no overlap in the 95% intervals indicates significant between means.

For all 290 models, the lagged EVI was significant for both the connected and controlled lakes (*p* < 0.001, Table 1). The mean temporal autocorrelation coefficient for connected sites was 0.39, significantly lower than those for the controlled lakes (0.47) (adjusted *p* < 0.001) (Table 1, Figure 6), suggesting the significantly lower resilience for wet meadows developed in hydrologically controlled lakes.

**Table 1:** Summary of the 290 AR models.

	**Connected (*****n*** = **165)**		**Controlled (*****n*** = **125)**	
	**Estimate (mean and range)**	***p* (range)**	**Estimate (mean and range)**	***p* (range)**
Water level	−0.19 (−0.33, −0.10)	<0.001–0.089	−0.16 (−0.24, −0.08)	<0.001–0.137
Rainfall	−0.13 (−0.24, −0.18)	<0.001–0.146	−0.12 (−0.20, −0.09)	<0.001–0.168
Lagged EVI	0.39 (0.15, 0.48)	<0.001	0.47 (0.33, 0.68)	<0.001

Water level had negative efforts on EVI, and the effort was significant for the majority of time series (*p* ranged from <0.001 to 0.137, and 85% of models had *p*-value < 0.05, Table 1). The permutation *t*-test indicated that the difference between connected and controlled lakes was significant at 0.05 level (*p* = 0.002 with interval between 0.000–0.011, Figure S5), indicating that resistance of wet meadow to water level perturbance was significantly higher in controlled lakes.

As with water level, rainfall was also negatively related with EVI, and the relationship was significant for most models (*p*-value ranged from < 0.001 to 0.168, and about 65% of the models had *p*-value < 0.05, Table 1). The difference between connected and controlled lakes was inconclusive (*p* = 0.070 with interval of 0.042, i.e., significant to 0.113, i.e., insignificant, Figure 6).

The negative values for all βs (the estimates for water level and rainfall, Table 1) suggested that biomass or greenness (as represented by EVI) decreased with higher water level and greater rainfall. Furthermore, as the absolute values for water level was greater those for rainfall in the majority of cases (95%), water level was the more influential factor for EVI dynamics.

## Discussion and conclusion

In floodplains, ecological stability is closely associated with flood regimes (Colloff and Baldwin, 2010), which in term are driven primarily by the flow regimes of the associated rivers (Opperman et al., 2009). Floods bring sediments and other materials, and can determine considerably the presence and extent of wetlands, the type, extent and density of vegetation, richness and diversity of wildlife (Bayley, 1995; Ward et al., 1999). In the middle Yangtze region, China, the trend toward earlier water level withdrawal (Zhang et al., 2014), drier winter seasons (Guan et al., 2014; Zhang et al., 2014), has been associated with the expansion of emergent vegetation (mainly *Carex* sedge, but also reeds *Phragmites australis* and canary grass *Phalaris arundinacea*) (Mei et al., 2016; Jing et al., 2017). The changes in vegetation community distribution and phenology have caused the shifts in foraging patterns and behaviors of wintering migratory waterbirds (Jia et al., 2013; Guan et al., 2016; Xia et al., 2016), promoting heated debates on the impacts of river regulation on floodplain ecological health (e.g., Zhang et al., 2012, 2014; Xia et al., 2016; Han et al., 2017).

In this study, we compared the ecological stability of wet meadows between controlled and connected lakes in a large seasonal floodplain in terms of resilience and resistance to environmental perturbations using the long-term (2000–2016) MODIS EVI time series. The main finding of our study was that the resilience of wet meadows was significantly higher in the more natural freely-connected lakes in comparison with the partially-controlled ones, i.e., wet meadows in the more natural habitats tend to return significantly faster to their average state after disturbances. However, the controlled lakes had significantly higher resistance to environmental anomalies, in particular, to water level. In a study of European terrestrial grasslands, De Keersmaecker et al. (2016) found that the semi-natural grasslands were less resilient but more resistant to environmental disturbance than agricultural grasslands. They attributed the higher resistance of semi-natural grasslands to the mediating role of species diversity as more diverse communities are likely to have higher probability that one or more species are resistant to a disturbance (i.e., “sampling effect theory,” Tilman et al., 1997) and niche “complementarity” (Caldeira et al., 2005), i.e., species differ spatially and temporally in resource requirements. Although the wet meadow in our study is largely monospecies (i.e., *Carex* spp.), these hypotheses might still be applicable. The higher resilience in natural lakes may be associated with higher diversity of sub-species, or the functional traits of the species (e.g., clonal reproduction versus seedling recruitment, Deng et al., 2015). The encroachment of *P. australis* and *P. arundinacea* in area with slightly higher evaluation (Jin and Lei, 2015) may have also contributed to the higher resilience. Sand mining in Poyang, particularly at the outlet, has greatly increased the channel discharging capacity (Lai et al., 2014), and has lowered winter water level in freely connected lakes. These changes in hydrological regime and lake bed morphology (Gao et al., 2014) facilitates the encroachment and development of mix grass patches on previous mudflats.

The management practices of the partially control lakes might contribute to the higher resistance. In the middle Yangtze floodplain, *Carex* typically has two growth seasons (Guan et al., 2014, Figure S4). In the regulated lakes, water level is kept artificially high and stable only during the first growth season (i.e., late September to late December). Therefore, the higher water level could suppress the first growth peak at early November but not the second one at early March, which is more dominant (Figure S4). In addition, the artificially high water level prohibits new seedling recruitment and lower the encroachment.

There were two circumstantial evidences suggested that the wet meadow patches in freely connected sub-lakes or flats might be approaching the threshold or near the tipping point (Andersen et al., 2009), or be undergoing regime shift (Biggs et al., 2009). First, the annual variation in EVI time series (seasonality) displayed a continuous increasing trend may be the early warning signal of regime change (Batt et al., 2013; Dai et al., 2015). When comparing the two growth cycles, the increase in the first peak (i.e., at November, Figure S4) is particularly faster and consistent (Figure S4) for the connected sites, suggesting the earlier water withdrawal due to the operation of the TGD and the enhanced channel capacity were the likely cause (Guan et al., 2014; Mei et al., 2016) rather than the height of water level, which showed an increasing trend after 2006 (Figure 3). Second, the increasing trend in EVI at connected lakes was de-coupled from the trends in environmental variables (both water level and rainfall, Figure 4). The de-coupled relationships gave a strong indicator of regime shifting (Hughes, 2012; Skalak et al., 2017). Further studies closely monitoring the expansion of *Carex* wet meadow into shallow waters, re-zonation of vegetation community, and the field based experiment on the interspecific interaction between dominated aquatic plants, would help to verify (or refute) the regime shift suggested by our results.

The persistent high productivity/biomass at connected lakes and unrestricted flats might have changed the distribution of waterbirds within Poyang Lake (Xia et al., 2016). For example, Siberian crane and oriental white stork, which feed mainly on the tubers and roots of submerged aquatic plant such as *Vallisneria spiralis*, are observed congregating in a few controlled lakes (Jia et al., 2013; Xia et al., 2016). The plausible regime shift in wet meadow at connected lakes and flats demands urgent and practical management actions to safeguard the long-term survival of the wintering avian species. On regional scale, adjustments to the current TGD operation to minimize the impacts on vegetation and wintering waterbird phenology, such as the timing and rate of autumn water recession (Guan et al., 2016) and summer water level rising (Wang et al., 2013), more natural water level fluctuations (Aharon-Rotman et al., 2017), are key adaptive management instruments. At site scale, regulating sand mining, such as the location and production, to stabilize the channel capacity and lake bed (de Leeuw et al., 2010; Lai et al., 2014), is also important. However, our results clearly discredited the proposal of construction of water level regulation weir at the outlet channel (Wang et al., 2015) because ecological resilience was significantly lower in controlled sub-lakes than in connected ones.

To conclude, we found that the resilience of floodplain wet meadows, as defined by the return of productivity response following perturbation, decreases with artificial hydrological modification. The higher resilience in the free-connected lakes enables the persistent and continuous increase in productivity and biomass of wet meadows in areas previously dominated by submerged macrophytes and/or mudflats. We linked the higher resilience in more natural environment with a diversity of sub-species or functional traits and the encroachment of *P. australis* and *P. arundinacea* facilitated by earlier water level withdrawal and lower winter water level. The higher resistance in controlled lakes might be ascribed to management practices which keep water level in those lake stable and high during the first growth cycle. Our results also indicated that the wet meadows in freely-connected lakes and flats might approach a tipping-point or be undergoing a regime shift. Regional and site scale management actions are proposed to avoid the imminent catastrophic impacts on migratory waterbirds, such as Siberian crane and oriental white stork, whose primarily feed ground is mudflats and shallow waters.

## Author contributions

The ideas of the paper were generated by discussions by all authors. LS compiled the EVI data; YW, YJ, and CL provided the environmental data. LS and LW did the data analysis. LW and LS wrote the first draft of the manuscript with contribution from others.

### Conflict of interest statement

The authors declare that the research was conducted in the absence of any commercial or financial relationships that could be construed as a potential conflict of interest.
