# Supplementary material for: Vegetation Cover Dynamics and Resilience to Climatic and Hydrological Disturbances in Seasonal Floodplain: The Effects of Hydrological Connectivity
Source: Front Plant Sci. 2017 Dec 22;8:2196. doi: 10.3389/fpls.2017.02196 (PMC5744444; doi:10.3389/fpls.2017.02196)
Supplement: Supplementary file 1 [file DataSheet1.docx]

**Appendix**

**
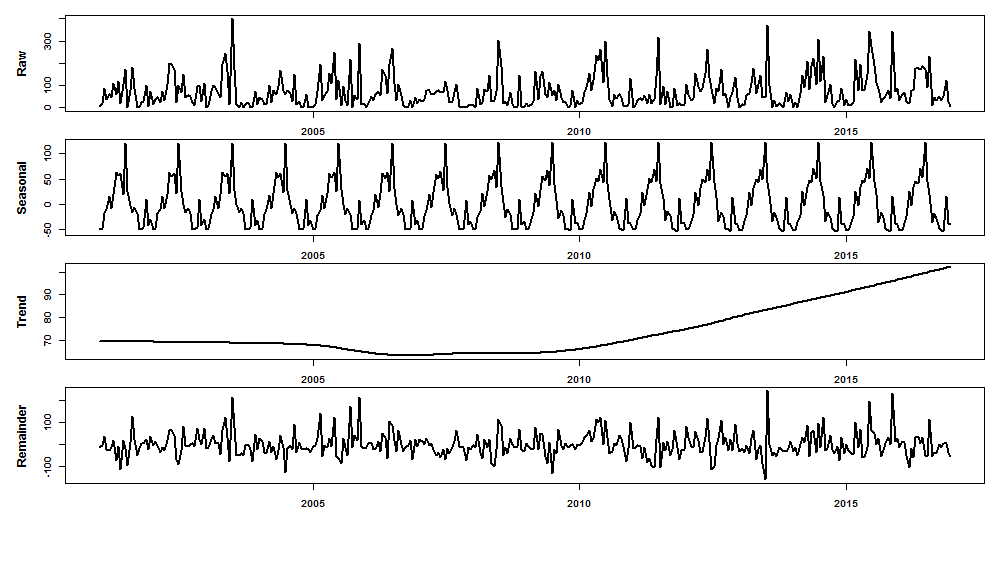
**

Figure S1 Time series of rainfall at Nanchang showing the clear seasonality and trend during the study period (2000 – 2016). A clear increasing trend is evident for 2010-2016.

**
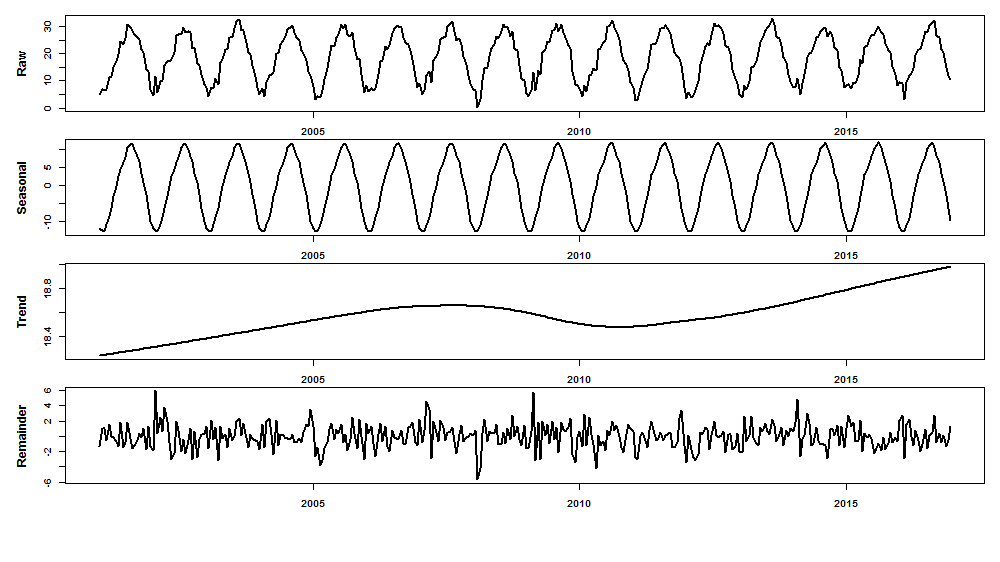
**

Figure S2 Time series of temperature at Nachang showing the clear seasonality and trend during the study period (2010 – 2016).


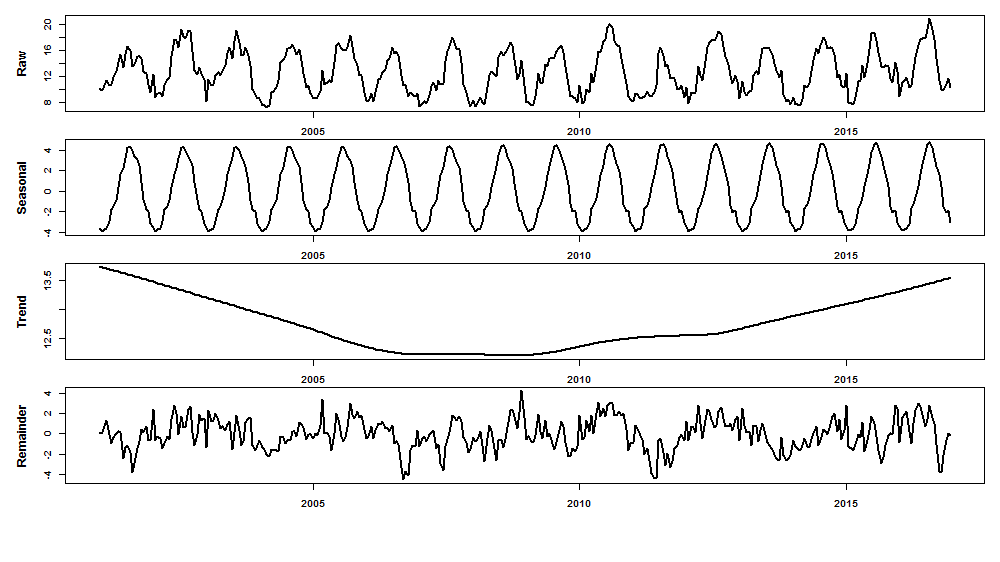


Figure S3 Water level at Xingzi showing the clear seasonality and trend during the study period (2000 – 2016), and the decomposed seasonality, trend and remainder. Data for climatic variables can be found in the appendix Figure 1S and 2S.


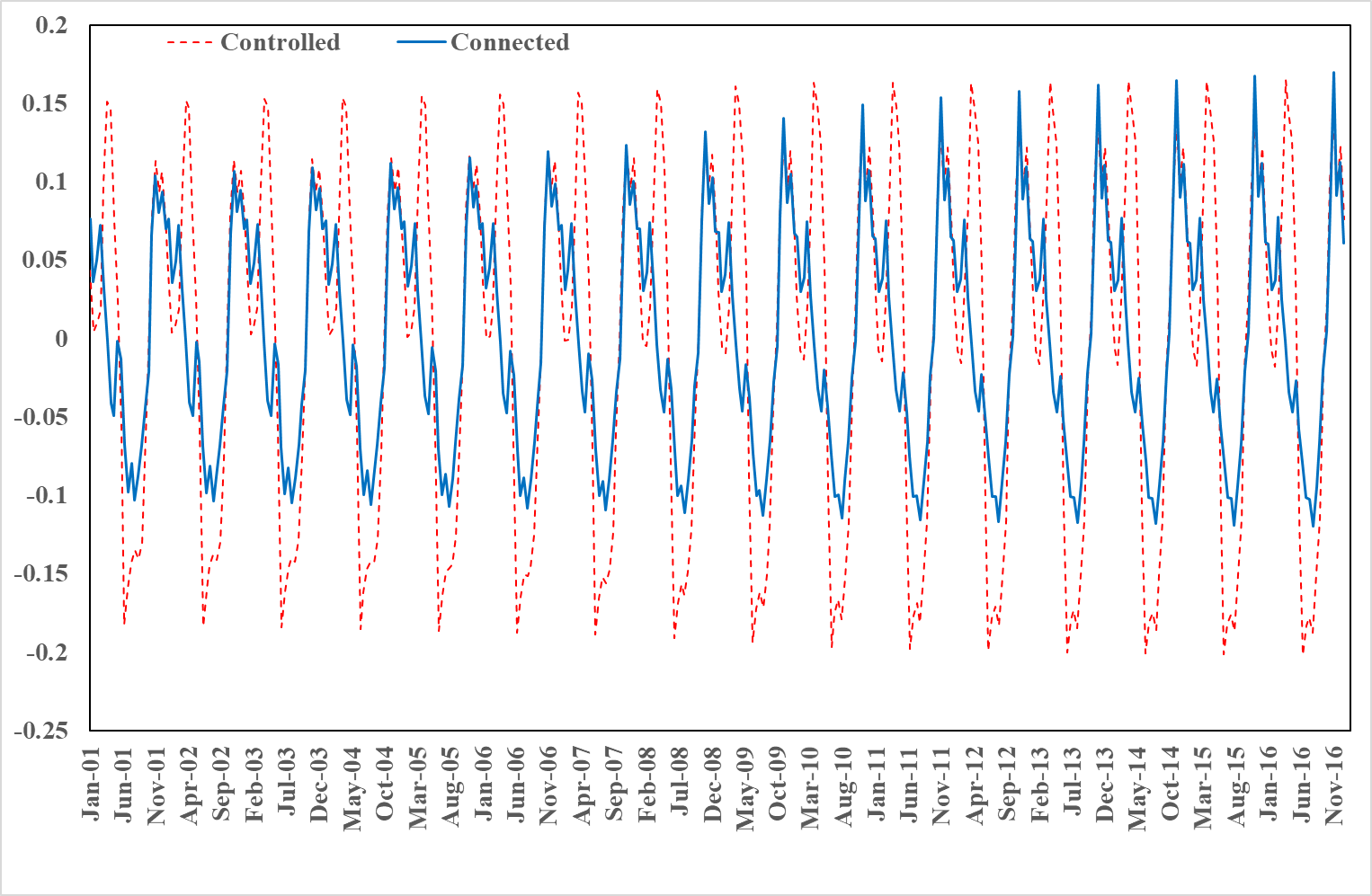


Figure S4 Examples of modelled seasonality of EVI time series in controlled and connected lakes. The two growth circles peak at November and March are typical for *Carex* in middle Yangtze region. The seasonality in connected lakes showed a continuous increasing trend, especially the first peak at November, but was relatively stable in controlled ones.


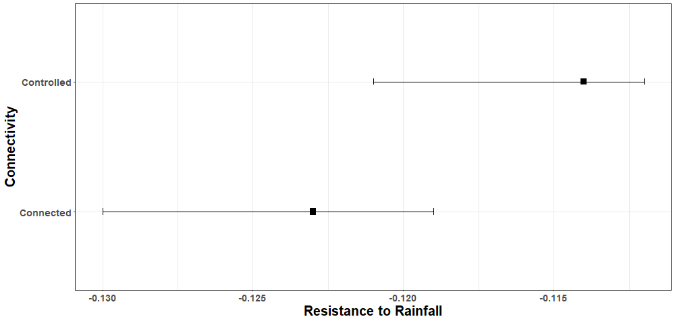

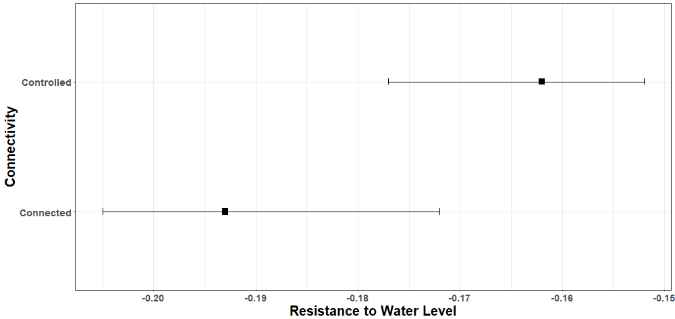


Figure S5 Comparison of resistance to rainfall and water level anomaly of wet meadows in connected and controlled sub-lakes. Squares are mean and bars are 95% confident intervals based on 10,000 bootstrap resampling. There is overlap in the 95% intervals indicates non-significant difference between means.
